# Alopecia in a Viable Phospholipase C Delta 1 and Phospholipase C Delta 3 Double Mutant

**DOI:** 10.1371/journal.pone.0039203

**Published:** 2012-06-19

**Authors:** Fabian Runkel, Maik Hintze, Sebastian Griesing, Marion Michels, Birgit Blanck, Kiyoko Fukami, Jean-Louis Guénet, Thomas Franz

**Affiliations:** 1 Anatomisches Institut, Universität Bonn, Bonn, Germany; 2 Studiengang Molekulare Biomedizin, LIMES, Bonn, Germany; 3 Laboratory of Genome and Biosignal, Tokyo University of Pharmacy and Life Science, Hachioji-city, Tokyo, Japan; 4 Département de Biologie du Développement, Institut Pasteur, Paris, France; Ohio State University Medical Center, United States of America

## Abstract

**Background:**

Inositol 1,4,5trisphosphate (IP_3_) and diacylglycerol (DAG) are important intracellular signalling molecules in various tissues. They are generated by the phospholipase C family of enzymes, of which phospholipase C delta (PLCD) forms one class. Studies with functional inactivation of *Plcd* isozyme encoding genes in mice have revealed that loss of both *Plcd1* and *Plcd3* causes early embryonic death. Inactivation of *Plcd1* alone causes loss of hair (alopecia), whereas inactivation of *Plcd3* alone has no apparent phenotypic effect. To investigate a possible synergy of *Plcd1* and *Plcd3* in postnatal mice, novel mutations of these genes compatible with life after birth need to be found.

**Methodology/Principal Findings:**

We characterise a novel mouse mutant with a spontaneously arisen mutation in *Plcd3* (*Plcd3^mNab^*) that resulted from the insertion of an intracisternal A particle (IAP) into intron 2 of the *Plcd3* gene. This mutation leads to the predominant expression of a truncated PLCD3 protein lacking the N-terminal PH domain. C3H mice that carry one or two mutant *Plcd3^mNab^* alleles are phenotypically normal. However, the presence of one *Plcd3^mNab^* allele exacerbates the alopecia caused by the loss of functional *Plcd1* in *Del(9)olt1Pas* mutant mice with respect to the number of hair follicles affected and the body region involved. Mice double homozygous for both the *Del(9)olt1Pas* and the *Plcd3^mNab^* mutations survive for several weeks and exhibit total alopecia associated with fragile hair shafts showing altered expression of some structural genes and shortened phases of proliferation in hair follicle matrix cells.

**Conclusions/Significance:**

The *Plcd3^mNab^* mutation is a novel hypomorphic mutation of *Plcd3*. Our investigations suggest that *Plcd1* and *Plcd3* have synergistic effects on the murine hair follicle in specific regions of the body surface.

## Introduction

Phosphoinositide metabolism provides an essential intracellular signalling system involved in a broad spectrum of key events in organ development and function. Phosphoinositol 4,5 bisphosphate (PIP_2_) is converted by phosphoinositide-specific phospholipase C (PLC) to inositol 1,4,5 trisphosphate (IP_3_) and diacylglycerol (DAG), which function as second messengers in the cell. DAG activates protein kinase C, and IP_3_ causes the release of calcium ions from intracellular stores, which makes PLC enzymes key regulators of intracellular calcium [1]–[11]. The starting material of this reaction, PIP_2_, is a signalling mediator in its own right involved in a variety of processes such as phagocytosis, ion channel activity and cell motility [2], [12].

PLC isozymes form six 6 classes based on their functional protein domains [10], [13]. Of these, the PLC delta isoform family (PLCD) appears to be the most basic form containing a pleckstrin homology (PH) domain, catalytic X and Y domains, EF hand domains and a C2 domain [13]–[18]. Three murine PLCD isozymes are known to date: PLCD1, PLCD3 and PLCD4 [13]–[15], [17]–[19]. The PH domain targets PLCD proteins to PIP_2_ in the plasma membrane and induces activating conformational alterations of the catalytic domain, which is essential for PLCD function [13], [20]–[29]. The activity of PLCD1 is also regulated by the interaction with other proteins such as calmodulin and small GTPases [30]–[32], and through site specific phosphorylation by protein kinase C alpha (PKC alpha) [29]. Some PLCD proteins can translocate to the nucleus and PIP_2_ derivatives play important roles in nuclear function [3], [5]–[7], [33], [34].

The in vivo role of PLCD isozymes has been studied in mice with functional inactivation of *Plcd1*, *Plcd3* and *Plcd4*, respectively. The lack of PLCD4 activity in genetically ablated mice causes disturbances of liver regeneration and interferes with the acrosome reaction in spermatozoa [35], [36], while over-expression of *Plcd4* in a breast cancer cell line induces anchorage-independent growth [37]. In contrast, the loss of the *Plcd3* gene, which is located on mouse chromosome 11, causes no phenotype suggesting that the lack of this enzyme can be compensated for [17], [38], although knockdown experiments in vitro and in vivo provided evidence for an involvement of PLCD3 in cortical and cerebellar neuronal migration and neurite formation [39]. In humans, down-regulation of PLCD3 in the right ventricular outflow tract may be associated with idiopathic ventricular arrhythmias [40] and a genomic locus associated with hypertension has been mapped near the PLCD3 locus [41]. *Plcd3* is expressed in various human tissues and is regulated in permanent cell lines by alterations of intracellular cAMP and calcium levels [42], [43].

Studies of mice with spontaneous or engineered ablation of the *Plcd1* gene, located on mouse chromosome 9, have revealed important roles for this PLC delta isozyme in the normal development and function of the skin and its appendages [44]–[47]. The *Del(9)olt1Pas* (synonym *Del(9Ctdspl-Slc22a14)1Pas*) mutation is the spontaneously arisen genetic defect of a recessive mouse mutation formerly called oligotriche (*olt*), which shows a combination of alopecia and male infertility. Although the *Del(9)olt1Pas* mutation is a large deletion on chromosome 9 encompassing the genes *Ctdspl* (carboxy-terminal domain RNA polymerase II polypeptide A small phosphatase-like), *Vill* (villin-like), *Plcd1*, *Dlec1* (deleted in lung and esophageal cancer 1), *Acaa1b* (acetyl-Coenzyme A acyltransferase 1B, synonym thiolase B), and a part of *Slc22a14* (solute carrier family 22 member 14), the alopecia of the mutant has been attributed to the loss of *Plcd1* [47]. Mutant mice in which *Plcd1* expression was disrupted by targeted gene inactivation [45], [46] and *Del(9)olt1Pas* mutant mice show varying degrees of hair loss (alopecia) [47]. However, the disruption of both *Plcd1* and *Plcd3* causes prenatal death in mice due to vasculature defects in the placenta [38], suggesting that both genes may co-operate with each other at least during critical phases of development.

Here, we report on a spontaneous hypomorphic mutation of *Plcd3* caused by the insertion of an intracisternal A particle (IAP) genome [48], [49] into the *Plcd3* gene, which causes the predominant expression of a truncated PLCD3 protein lacking the PH domain. Mice homozygous for both the *Del(9)olt1Pas* deletion and the novel mutant *Plcd3^mNab^* gene live for several weeks after birth and show total alopecia.

The hair follicle is a highly complex and dynamic part of the integument which originates from stem cells and undergoes recurring phases of growth (anagen), regression (catagen) and rest (telogen), and produces the hair shaft which contributes to the pelage [50]–[56]. There are several hundred mutations in mice that cause phenotypic alterations in the pelage [51], which has been pivotal in understanding the molecular mechanisms of hair follicle growth and differentiation. In this report, we show that the phases of growth and regression in hair follicles of the dorsal skin are dramatically altered in all hair follicles of mice homozygous for both the *Del(9)olt1Pas* deletion and the novel mutant *Plcd3^mNab^* gene.

## Results

### Origin of *oltSH* and *oltNH* Mice

**Figure 1:**
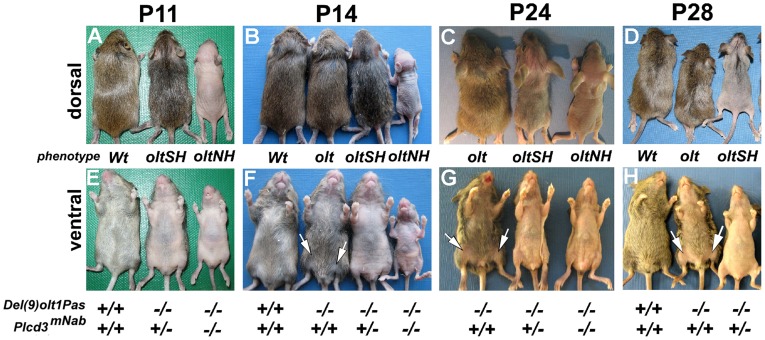
The phenotypes of wild-type, *Del(9)olt1Pas, oltSH* and *oltNH* mutant mice. The phenotype of wild-type (Wt), *Del(9)olt1Pas* (*olt*) homozygotes, *oltSH* and *oltNH* mice during hair follicle morphogenesis (P11 and P14) and the first hair cycle (P24 and P28). The + and – indicate the wild-type and mutant allele, respectively. The genotypes given below the images were determined by genomic PCR assays as described in Figure 2 B. Mutants of the *oltSH* phenotype have a reduced dorsal pelage on day P11 and P14 (A and B), which becomes very sparse during the first hair cycle (C and D). Ventrally, *oltSH* mutants show total alopecia (E to H) compared to homozygous *Del(9)olt1Pas* mice (F to H), in which the coat is predominantly reduced in the medial femoral and inguinal region (arrows in F, G, and H). *oltNH* mutants have no pelage.

A novel pelage phenotype, which we provisionally called *oltSH* (*SH* for sparse hair), occurred spontaneously in our stock of *Del(9)olt1Pas* mutant mice (also see Figure S1). In phenotypic *oltSH* mice, the loss of pelage by far exceeded the alopecia of homozygous *Del(9)olt1Pas* mutant mice during hair follicle morphogenesis, which was most pronounced on the ventral surface (Figure 1A,B,E,F) [47]. While in *Del(9)olt1Pas* mutant mice on postnatal day 14 merely the inguinal and medial femoral region showed a pronounced loss of pelage (arrows in Figure 1F), the *oltSH* mutant was virtually hairless ventrally (Figure 1F). The loss of dorsal pelage in *oltSH* mice increased dramatically in the first cycle of hair growth on postnatal day 24, until by postnatal day 28 merely some hairs remained in the dorsal midline and the face (Figure 1C,D,G,H). Crossing of *oltSH* females with *Del(9)olt1Pas* heterozygous males yielded phenotypic offspring of both the *Del(9)olt1Pas* and the *oltSH* phenotypes in equal numbers, indicating that one dose of the novel mutant allele had caused the exacerbation of the phenotype of *Del(9)olt1Pas* homozygous mutants.

In order to determine the phenotype of mice being double homozygous for the novel *oltSH* mutation and the *Del(9)olt1Pas* mutation, we set up matings of *oltSH* females with phenotypically normal males descending from *oltSH* females bred with *Del(9)olt1Pas* heterozygous males. Among the offspring, we found 10 mice that had not grown any visible pelage and only a few short vibrissae, which we provisionally called *oltNH* (*NH* for no hair) (Figure 1, Figure S1).

Apart from the total alopecia, *oltNH* mice were also smaller and weighed less than their littermates. On postnatal day 8, the body weight of wild-type mice was 5,7 gr ±0,6 gr (n = 6), of *oltSH* mice 4,9 gr ±0,3 gr (n = 3), and of *oltNH* mice 3,8 gr ±0,1 gr (n = 3), and on postnatal day 25, wild-type mice weighed 11,5 gr ±1,7 gr (n = 4), *oltSH* mice 9,3 gr ±0,4 gr (n = 3), and *oltNH* mice 6,7 gr ±0,8 gr (n = 3). While *Del(9)olt1Pas* homozygous mutants and *oltSH* mice have lived for more than 1 year, almost no *oltNH* mouse has lived longer than 40 days (n = 21) (with one exception of 64 days). Thus, the combination of the *Del(9)olt1Pas* mutation with the novel mutation had marked additive effects on the dorsal pelage, the body mass and the longevity.

### The *oltSH* and *oltNH* Phenotypes are Associated with Altered *Plcd3* Transcripts

Since we had previously identified that the *Del(9)olt1Pas* pelage phenotype was caused by the lack of expression of the *Plcd1* gene [47], we examined whether the mutation causing the *oltSH* and *oltNH* phenotypes might involve other members of the *Plcd* gene family.

**Figure 2:**
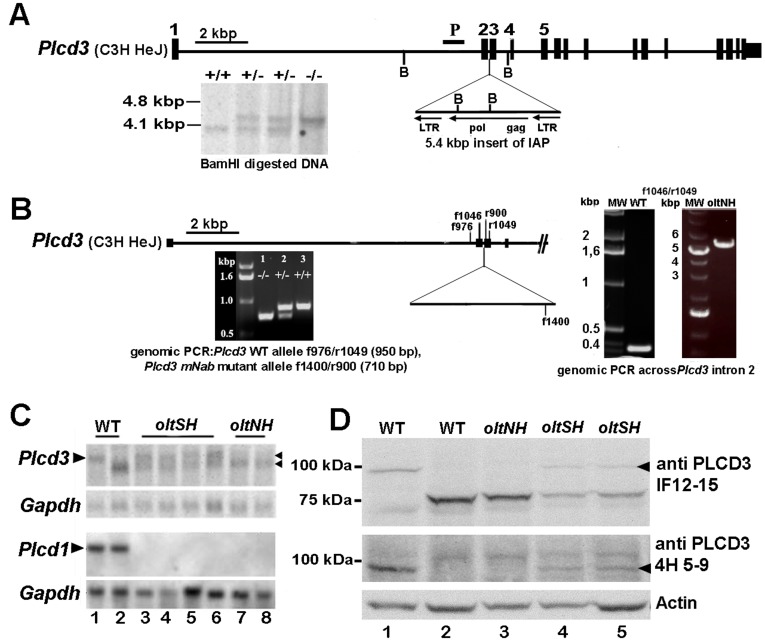
Molecular analysis of the *Plcd3* ^mNab^ mutation. A. Schematic representation of the genomic context of the *Plcd3^mNab^* mutation. The *Plcd3^mNab^* mutation is caused by the insertion of 5.4 kbp of IAP sequences in intron 2 of *Plcd3*. Hybridisation with a probe (P) derived from intron 1 using primers 572 and 573 (Table S1) reveals a restriction fragment length polymorphism in BamHI digested genomic DNA of wild-type (+/+), *oltSH* (+/−) and *oltNH* (−/−) mice, respectively. The wild-type allele shows a 4.1 kbp fragment and the mutant allele a fragment of 4.5 kbp. The increased length of the mutant fragment is caused by the BamHI site (B) within the inserted IAP. The *Plcd3* locus is shown in reverse orientation with respect to the chromosomal DNA, with *Plcd3* exon 1 to the left. The intracisternal A particle (IAP) has no *env* sequences and is inserted in intron 2 in reverse orientation with respect to the transcription of the *Plcd3* gene. B. Location of the primers used to analyse the *Plcd3^mNab^* mutant and the wild-type *Plcd3* locus (f, forward; r, reverse). On the left, electrophoresis of amplified genomic DNA fragments that are indicative of the wild-type *Plcd3* and the *Plcd3^mNab^* allele using the primers indicated. On the right, the PCR products from genomic DNA around *Plcd3* intron 2 obtained from wild-type and *oltNH* mice using primers f1046 and r1049 (Table S1) are shown. The 5.4 kbp long fragment in the *oltNH* mutant contains the inserted IAP sequence. C. Northern blot analysis of mutant dorsal skin. A DIG-labelled probe derived from the 5′ region of *Plcd1* hybridises to transcripts only in phenotypically wild-type animals. A DIG-labelled RNA probe derived from the 3′ region of *Plcd3* hybridises to two transcripts of 3 kb and 2.6 kb, respectively (marked by arrowheads). *oltSH* mice express both transcripts, while *oltNH* mice show only the 2.6 kb transcript. Note that the wild-type mouse in lane 2 shows the mutant *Plcd3* transcript and expresses *Plcd1*. Hybridisation with a *Gapdh*-specific probe as loading control is also shown. D. Western blot analysis of mutant dorsal skin using antibody IF12–15 directed against the catalytic region of PLCD3 protein and 4H 5–9 directed against the PH domain of PLCD3. The phenotypes of the mice (Wt, *oltNH*, *oltSH*), from which the lysates were obtained, are given on top of each lane. The 2.6 kb mutant *Plcd3* transcript is translated to a truncated protein of 75 kDa, which is detected by the antibody IF12–15, but not the PH domain-specific antibody 4H 5–9 (lanes 2 and 3). This antibody also bound to an unknown protein of 110 kDa in all samples. Arrowheads indicate the wild-type protein. An immunoblot using antibody against actin is given below as a control. Note that the phenotypically wild-type in lane 2 is the same as the one in lane 2 of the Northern blot in C.

Southern blot analyses of BamHI digested genomic DNA from wild-type, *oltSH* and *oltNH* mice hybridised with a probe derived from *Plcd3* intron 1 (Figure 2A) revealed a restriction fragment length polymorphism (RFLP) showing the expected 4.1 kbp fragment in C3H wild-type DNA (based on the Celera mouse genome sequence), and a 4.5 kbp fragment in *oltNH* genomic DNA (Figure 2A).

As this BamHI fragment in wild-type genomic DNA of C3H mice stretches from intron 1 to intron 3 of *Plcd3*, we attempted to generate short genomic fragments for sequence analyses from this area by PCR using wild-type (C3HeB/FeJ) and mutant *oltNH* genomic DNA. Using primers 1046 of *Plcd3* exon 2 and 1049 of exon 3, we amplified the expected 320 bp fragment from wild-type genomic DNA, but a 5.4 kbp fragment from *oltNH* genomic DNA (Figure 2B). The genomic DNA sequence of this 5.4 kbp fragment revealed the insertion of an intracisternal A particle (IAP) into intron 2 of *Plcd3* (Figure 2A). The IAP contains flanking LTRs and a gag-pol region, but no env sequences, is oriented opposite to the transcription of the *Plcd3* gene, and is in its entire length 98% homologous to an IAP recently described (gb|FJ854359.1|) [57]. The RFLP observed in BamHI digested *oltSH* genomic DNA was thus caused by the insertion of a BamHI site within the IAP sequences (Figure 2A). We refer to this mutation as the *Plcd3^mNab^* mutation (*Nab* for Neuroanatomy Bonn).

Based on the sequence data, we designed a genomic PCR screen to identify the *Plcd3^mNab^* mutation in mice (Figure 2B). In combination with the PCR screen to identify the *Del(9)olt1Pas* mutation [47], we have so far analysed 246 mice in 37 litters and consistently found that the genotype of phenotypically *oltSH* mice was (*Del(9)olt1Pas*) −/−, *Plcd3^mNab^* +/− (n  = 21), while that of phenotypically *oltNH* mice was (*Del(9)olt1Pas*) −/−, *Plcd3^mNab^* −/− (n  = 24), with + referring to the respective wild-type allele. We also identified 8 phenotypically normal *Plcd3^mNab^* −/− mice that had at least one wild-type allele of *Plcd1*.

Northern blot analyses of total RNA obtained from dorsal skin demonstrated that mice of the *oltSH* or *oltNH* phenotype lacked expression of *Plcd1* mRNA (Figure 2C) and expressed truncated *Plcd3* transcripts (Figure 2C). While *oltSH* mutants expressed both the wild-type and the truncated *Plcd3* transcripts, *oltNH* mutants expressed only the truncated *Plcd3* transcript.

We also performed Western Blot analyses on protein lysates obtained from dorsal skin using monoclonal antibodies binding specifically either to the N-terminal PH domain of PLCD3 (4H 5–9) or to the more centrally localised catalytic domain (IF12–15) (Figure 2D). The antibody directed against the PH domain detected the 88 kDa full-length PLCD3 protein in wild-type and *oltSH* skin lysates, but showed no corresponding band in lysates from *Plcd3^mNab^* homozygous mice. The antibody directed against the catalytic domain detected the 88 kDa band in wild-type mice, and a band of approximately 75 kDa in *oltNH* mice. In the *oltSH* mutant, both the 88 kDa and the 75 kDa protein were found (Figure 2B). These experiments suggest that the *Plcd3^mNab^* mutation contributed to the *oltSH* and *oltNH* phenotypes by expressing a truncated PLCD3 protein variant that lacks the PH domain.

Altered *Plcd3* transcripts and PLCD3 protein were also found in phenotypically wild-type mice (example in lane 2, Figure 2C and D) that expressed *Plcd1* normally, confirming that the expression of an altered *Plcd3* transcript is not sufficient to cause a phenotype by itself, but only in combination with the loss of *Plcd1* expression [38].

Thus, *Plcd3* is not required for normal hair follicle morphogenesis in dorsal skin in the presence of at least one wild-type allele of *Plcd1*, but truncation of PLCD3 in *Plcd1*-defective mice aggravates the loss of dorsal pelage suggesting that *Plcd3* expression may compensate at least partially for the loss of *Plcd1* expression in dorsal hair follicles.

### Hair Follicle Morphology of *oltSH* and *oltNH* Mutant Mice

**Figure 3:**
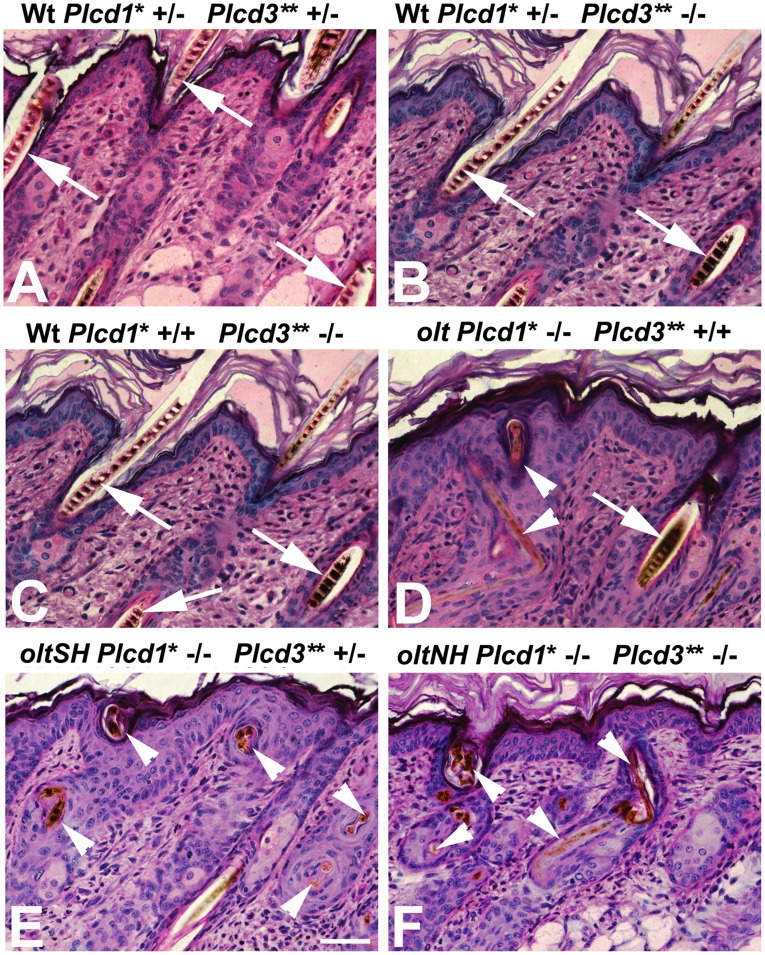
Histology of the infundibular region and distorted hair shafts. Methacrylate (Technovit 7100) sections of representative areas in the dorsal skin on postnatal day 9, HE stain. The phenotype (Wt, *olt* (i.e. *Del(9)olt1Pas*), *oltSH* or *oltNH*, respectively) and genotype with respect to the *Plcd1* (Plcd1* “-” refers to the *Del(9)olt1Pas* mutation) and *Plcd3* (Plcd3** “-” refers to the *Plcd3* ^mNab^ allele) gene is indicated for each image. At least 4 biopsies of each genotype have been investigated. In E, Bar  = 25 µm. These is no hair loss and are no distorted hair shafts neither in wild-type mice heterozygous for both mutant alleles (A), nor those heterozygous for the *Del(9)olt1Pas* mutation and homozygous for the *Plcd3* ^mNab^ allele (B), nor others being wild-type for *Plcd1* and homozygous for the *Plcd3* ^mNab^ mutant allele (C). Arrows indicate normal hair shafts. Arrowheads mark distorted hair shafts in *Del(9)olt1Pas* (*olt*), *oltSH* and *oltNH* mice. The alterations of the hair shafts appear histologically similar in all three mutant specimens.

The absence of visible pelage in *oltNH* mice and its sparseness in *oltSH* mutants is not caused by the absence of hair follicles, but the disability of the hair shafts to penetrate to the surface. On postnatal day 9, wild-type (Wt) hair shafts are straight and penetrate through the pilary canal to the surface, irrespective of being heterozygous (arrows in Figure 3A) or homozygous (arrows in Figure 3B,C) for the *Plcd3^mNab^* mutation. However, distorted hair shafts in the *Del(9)olt1Pas* (Figure 3D, “*olt*”), the *oltSH* (Figure 3E) and the *oltNH* (Figure 3F) mutants are bent and curled either within the pilary canal or underneath the stratum corneum (arrowheads in Figure 3D,E,F). While the histological aspects of this distortion did not differ notably among the three different mutants, the percentage of dorsal hair follicles affected did vary. In *Del(9)olt1Pas* mutants 13,5±3% of hair shafts were distorted in the dorsal skin, while in *oltSH* mutants 66±5,8% of hair shafts were affected, whereas in *oltNH* mutants all hair shafts were distorted failing to penetrate to the surface (150 hair follicles counted in 3 different biopsies for each mutant). Thus, in the absence of *Plcd1* expression, an increasing dosage of the *Plcd3^mNab^* mutation causes more dorsal hair shafts to be defective.

### Histological Examination of the Hair Follicle Morphogenesis and First Cycle in *oltNH* Mutants

**Figure 4:**
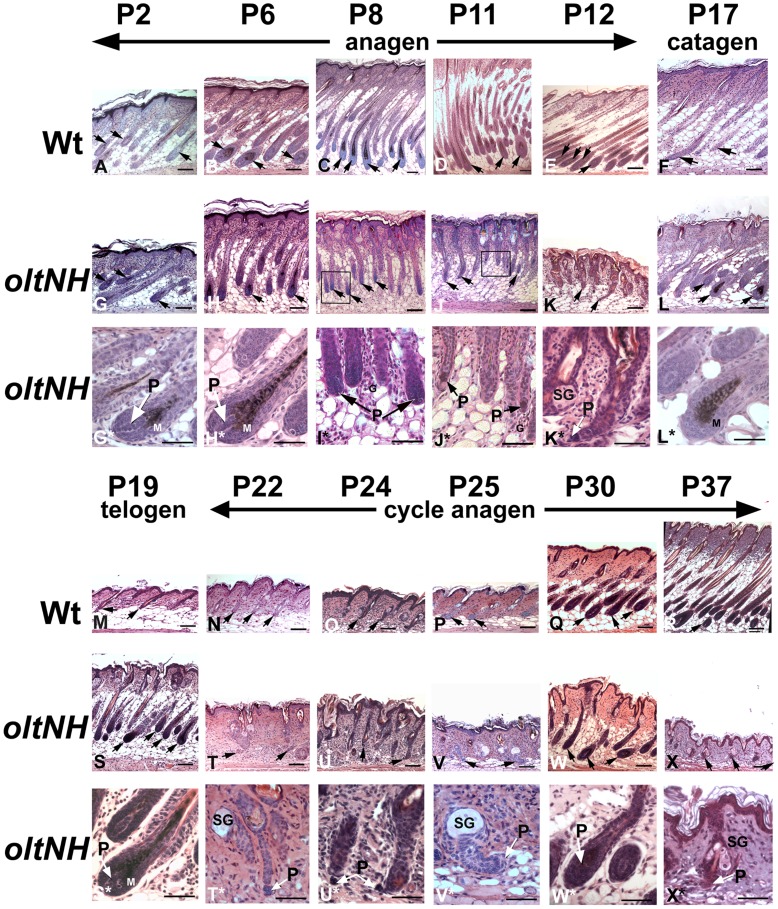
Histology of wild-type and *oltNH* hair follicles during hair follicle morphogenesis and the beginning of the first cycle. Paraffin and methacrylate (Technovit 7100) sections, HE stain. Postnatal days examined are indicated. In wild-type animals, the hair follicles increase in length during anagen from P2 to P12 and show active melanogenesis in their hair bulbs during this period (arrows in A to E). The diameter of the hair bulb decreased from P6 to P11 and remained like this until catagen sets in on postnatal day 17 (F). In *oltNH* mice, however, the hair follicles decrease in length after postnatal day 6 (G to K) and exclude the dermal papilla from the bulb on days 11 and 12 (arrows P in J* and K*). Numerous granulocytes (G) are found in the vicinity of the mutant hair bulbs at this time (marked as G in I* and J*). The diameter of the hair bulb decreases remarkably after postnatal day 6 (arrows in G to K). While wild-type mice have entered catagen by day 17 as shown by the long epithelial strand and reduced hair follicle length (arrows in F), *oltNH* hair follicles re-enter an anagen phase on days 17 to 19 (L and S) exhibiting a broad hair matrix (marked as M in L* and S*), a large dermal papilla (marked as P arrow in S*) and active melanogenesis (L* and S*), which is followed by a regression on postnatal days 22 (T and T*) and 24 (U and U*). While wild-type hair follicles proceed through the first cycle anagen from day 22 to day 37 (arrows in N to R), *oltNH* hair follicles re-enter anagen on postnatal day 25 (V, marked as P arrow in V*) and show continued increase in hair follicle length by day 30 (W and W*). This growth phase of the mutant follicle ends in a telogen phase on day 37 (X and X*), when wild-type follicles are still in the growth phase (R). Three biopsies from different animals were used for each time point investigated. P, dermal papilla; SG, sebaceous gland; M, matrix; G, neutrophilic granulocyes. Bar  = 100 µm in images A to X, and 50 µm in images G* to L* and S* to X*.

To analyse the stages of hair follicle morphogenesis and the first hair cycle, we investigated biopsies of dorsal skin in wild-type and *oltNH* mice histologically from postnatal day 2 to day 37. The wild-type specimens showed hair follicles in anagen stages from day 2 to day 12 (Figure 4A to E), in the catagen phase on day 17 (Figure 4F), followed by the resting phase, telogen, on day 19 (Figure 4M) and thereafter again anagen stages of the first hair cycle (Figure 4N to R). In *oltNH* mutants, however, the growth phase lasted only from day 2 to day 8 (Figure 4G to I), when the hair bulbs became visibly narrower (Figure 4I*), and the follicles were shorter than in the wild-type (compare Figure 4I to 4C). On postnatal days 11 and 12, the mutant hair follicles were further shortened (Figure 4J and K) and the dermal papilla had been excluded from the trailing epithelial end of the regressing follicle (Figure 4J* and K*). This premature regression of the mutant hair follicles was accompanied by the appearance of numerous granulocytes in the adjacent subcutaneous tissue (marked as G in Figure 4I* and J*). By day 17, *oltNH* mutants had re-entered a growth phase and showed anagen hair follicles still on day 19 (Figure 4L and S). After a further regression phase on days 22 and 24 (Figure 4T and U), in which the dermal papilla had been excluded again from the lower end of the hair follicle (Figure 4T* and U*), the mutant hair follicles entered into another growth phase by day 30 (Figure 4V, V*, W and W*), which ended in a telogen phase by day 37 (Figure 4X and X*).

**Figure 5:**
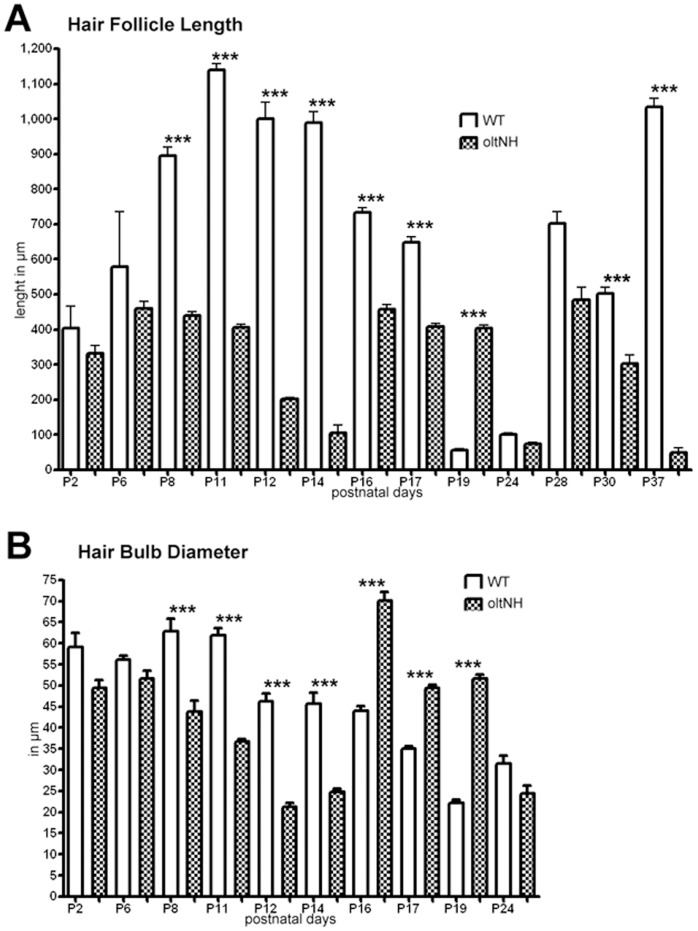
Histomorphometric analysis of hair follicle length and hair bulb diameter. Measurements were taken from a sample of 150 hair follicles in three different biopsies using the Image J software. Statistical analysis using the paired t-Test was performed employing the GraphPad Prism4 software. Data are expressed as mean ± SEM. *** signifies p<0.001. White columns depict data from wild-type mice and chequered columns those from *oltNH* mice. The age of the mice is given on the X axis. Hair follicle length represents the distance from the infundibulum to the most distal part of the hair follicle. The widest diameter of the hair bulb or distal end of the hair follicle in catagen and telogen stages is shown as “hair bulb diameter”. Both parameters indicate that *oltNH* mice terminate their first postnatal anagen by day 14 and re-initiate a growth phase thereafter, which in turn ends by day 24. The second cyclic growth phase of *oltNH* hair follicles ends by day 37.

We also quantified these observations measuring the hair follicle length (Figure 5A) and the width of the hair bulbs (Figure 5B). These measurements of hair follicle length corroborate that *oltNH* mutant hair follicles have completed three growth phases by day 37, when wild-type hair follicles are still in the middle of the second growth phase, i.e. the first cycle anagen (Figure 5A). They also show that after postnatal day 6, the width of the *oltNH* hair follicle bulb is significantly reduced compared to the wild-type (Figure 5B).

Thus, all hair follicles in *oltNH* mutants lacking functional *Plcd1* and expressing only the mutant *Plcd3^mNab^* allele show shortened phases of growth and regression that are not synchronous with the morphogenesis and cycle stages of hair follicles in wild-type mice.

**Figure 6:**
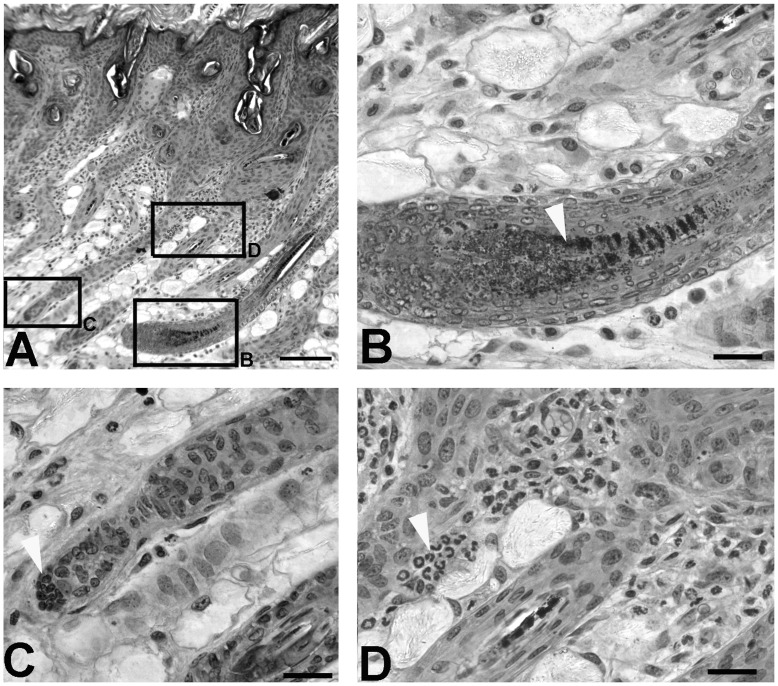
Histological aspects of *oltSH* mutants on postnatal day 12. Methacrylate (Technovit 7100) sections of representative areas in the dorsal skin on postnatal day 12 *oltSH* mice, HE stain, bar  = 100 µm in A, bar  = 25 µm in B to D. Boxed areas in A are shown at higher magnification B, C, and D respectively. Four different animals were investigated. A. The overview shows hair follicles in anagen (box B) and catagen (box C) side by side. B. The hair follicle shows active melanogenesis as a hallmark of anagen (arrowhead). C. The hair follicle is in catagen and has excluded the dermal papilla (arrowhead). D. Accumulations of neutrophilic granulocytes (arrowhead) are seen in the vicinity of follicles in catagen.

We also examined *oltSH* mutants histologically on postnatal day 12. When wild-type hair follicles were in anagen and *oltNH* mutant hair follicles were in regression (Figure 4E and K), *oltSH* exhibited hair follicles in anagen and regression side by side (Figure 6A to C). Inflammatory infiltrates consisting of neutrophilic granulocytes were found at the border between dermis and in the subcutaneous layer in the vicinity of regressing hair follicles (Figure 6D). As *Plcd3* is expressed in all hair follicles during anagen, the heterogeneity of hair follicle stages in *oltSH* mutants on day 12 compared to wild-type and *oltNH* mutants may suggest that the isolated premature entry into regression could possibly be caused by the limited concentration of unknown stimulating or negative factors exceeding a threshold for some, but not all hair follicles.

### Proliferation and Apoptosis in *oltNH* Hair Follicles

**Figure 7:**
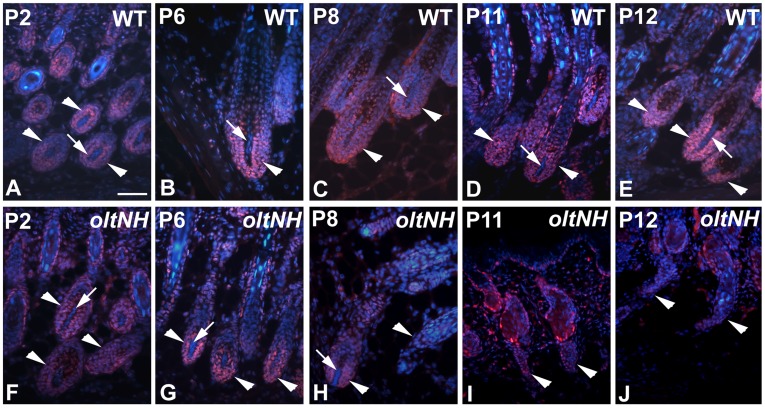
Proliferation in wild-type and *oltNH* hair follicles. Proliferation in the hair follicles of wild-type (A, B, C, D, E) and *oltNH* (*del(9)olt1Pas* −/−, *Plcd3^mNab^* −/−) (F, G, H, I, J) dorsal skin on postnatal day 2 (A, F), day 6 (B, G), day 8 (C, H), day 11 (D, I), and day 12 (E, J) is visualised by PCNA immunoreactivity (red signal). DAPI was used as a nuclear counter stain (blue signal). Paraffin sections. Bar  = 50 µm for all images is given in A. White arrowheads mark the hair bulb. Three biopsies from different animals were used for each time point investigated. In wild-type mice, PCNA immunoreactivity is detected in the nuclei of the matrix cells of the hair bulb throughout the period examined (arrowheads in A to E). In the *oltNH* mutant, the PCNA immunoreactivity is prominent in the matrix on days 2 and 6 (arrowheads in F and G), while already on day 8 some hair bulbs show much fainter immunoreactivity (right arrowhead in H). In the trailing ends of the hair follicles on days 11 the immunoreactivity is very faint (arrowheads in I), and on day 12 undetectable (arrowhead in J). Note that there is no PCNA immunoreactivity in the cells of the dermal papilla.

**Figure 8:**
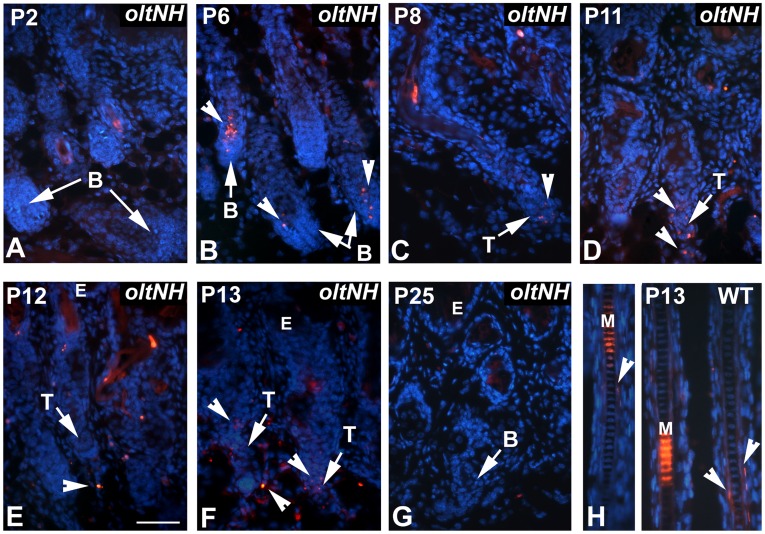
Apoptosis in *oltNH* hair follicles. Apoptosis was investigated by performing the TUNEL assay on paraffin sections. Apoptosis is visualised by Cy3 fluorescence (red signal). DAPI was used as a nuclear counterstain (blue signal). Sections were taken from skin biopsies of *oltNH* mice (A to G) from postnatal day 2 to 25. A wild-type skin section on postnatal day 13 is shown as control (H). Three biopsies from different animals were used for each time point investigated. Arrows mark the hair bulb (B) or the trailing ends (T) of regressing hair follicles. Arrowheads point at TUNEL positive cells. There are numerous TUNEL positive cells in the matrix of *oltNH* hair follicles beginning on days 6 and 8 (arrow heads in B and C) and the regressing follicles on day 11 to 13. On day 25, the *oltNH* hair follicle re-enters anagen and shows no TUNEL positive cells (arrow in G). Throughout this period, there were no TUNEL positive cells in the hair bulbs of wild-type mice (not shown), only some cells in the inner root sheath exhibited a TUNEL positive signal (arrowheads in H), while the medulla showed unspecific autofluorescence. E, epidermis. Bar in E  = 50 µm for all images.

As the histological investigation had suggested that *oltNH* hair follicles enter a phase of regression by postnatal day 8, we examined proliferation (Figure 7) and apoptosis (Figure 8) in mutant and wild-type hair follicles during this critical period.

Using PCNA immunoreactivity as a marker for proliferation, we found that cells in the matrix of wild-type hair bulbs proliferate from days 2 to 12 (Figure 7A to E). Similar PCNA immunoreactivity was detected in *oltNH* mutant hair bulbs from day 2 to day 8 (Figure 7F to H). While there was still some faint PCNA immunoreactivity in the histologically regressing hair follicles in the *oltNH* mutant on day 11 (arrowheads in Figure 7I), there was none left on day 12 (arrowheads Figure 7J). Thus, proliferation of precursor cells in the hair bulb of *oltNH* mutants does not continue beyond day 11.

Using the TUNEL assay as an indicator of apoptosis, we found no sign of apoptosis in the *oltNH* hair bulb during the early anagen stage of hair follicle morphogenesis on day 2 (Figure 8A) and the following anagen phase on day 25 (Figure 8G), while numerous TUNEL positive cells were found in the bulb region of mutant hair follicles on day 6 to 13 (arrowheads in Figure 8B to F). Throughout these stages examined in the *oltNH* mutants, hair bulbs of wild-type hair follicles showed no TUNEL positive cells (not shown), but some TUNEL positive cells in the inner root sheath (arrowheads in Figure 8H), which has been observed before [58]. Thus, in the *oltNH* hair matrix, cell proliferation and apoptosis co-exist from postnatal days 6 to 11, which may explain why the hair bulbs of the mutant are not the same size as those of the wild-type after day 6. These observations suggest that expression of *Plcd1* and *Plcd3* may be required for proliferation and survival of matrix cells in dorsal hair follicles.

### Expression of *Plcd3* During Hair Follicle Morphogenesis

**Figure 9:**
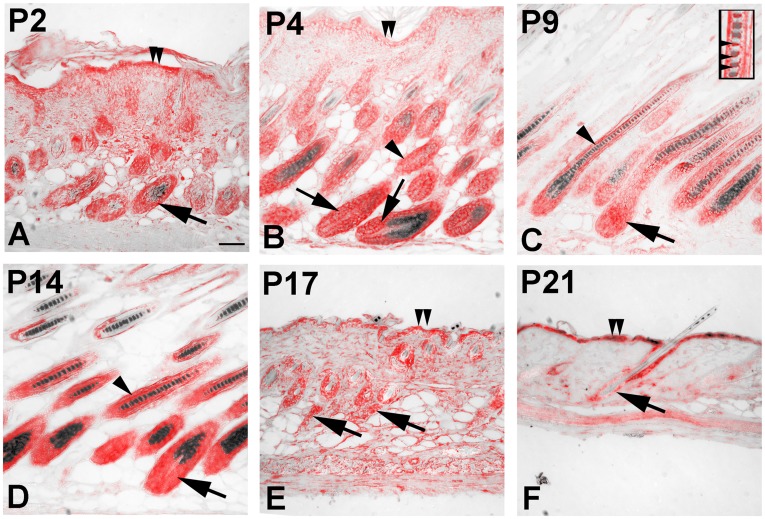
Expression of *Plcd3* in the murine hair follicle. Paraffin sections of skin biopsies obtained from C57BL/6J mice at the indicated age were hybridised with a probe derived from exons 3 to 5 of murine *Plcd3*. The DIG-labelled probe was visualised using alkaline phosphatase-conjugated anti DIG antibody. The stained sections were submitted to a spectral analysis using a Cri Nuance VX camera and software in the brightfield mode. The specific in situ signal spectrum is shown in red and the eumelanin signal in black. Three biopsies from different animals were used for each time point investigated. Bar in A  = 50 µm for all images. *Plcd3* is expressed in the hair bulb during anagen (arrows in A to D), in the trailing end during catagen (arrows in E) and during telogen in the epithelial cells covering the hair club (arrow in F). Expression of *Plcd3* is also found in the inner root sheath and cortex (arrowheads in B, C, D), the medulla (higher magnification insert in C) and the epidermis (double arrowheads in A, B, E, F).

To identify cell types and developmental stages possibly affected by the *Plcd3^mNab^* mutation in *oltSH* and *oltNH* mutants, we examined the expression of *Plcd3* during hair follicle morphogenesis in wild-type C57BL/6J mice using a probe covering *Plcd3* sequences from exon 3 to exon 5. *Plcd3* expression was found from postnatal days 2 to 14 in the hair bulb (arrows in Figure 9A to D), as well as in the trailing ends during catagen on day 17 (arrows in Figure 9E) and the cells surrounding the club hair during telogen (arrow in Figure 9F). *Plcd3* is also expressed in the inner root sheath and cortex (arrowheads in Figure 9B to D), the medulla (insert in Figure 9C) and the epidermis (double arrowhead in Figure 9A,B,E,F). We also detected expression in the dermis on day 2, day 4 and day 17 (Figure 9A,B,E). The expression of *Plcd3* in the hair matrix supports a possible involvement of *Plcd3* in regulating the proliferation and survival of these progenitor cells.

### Expression of Structural and Regulatory Genes in *oltNH* Mutants

**Figure 10:**
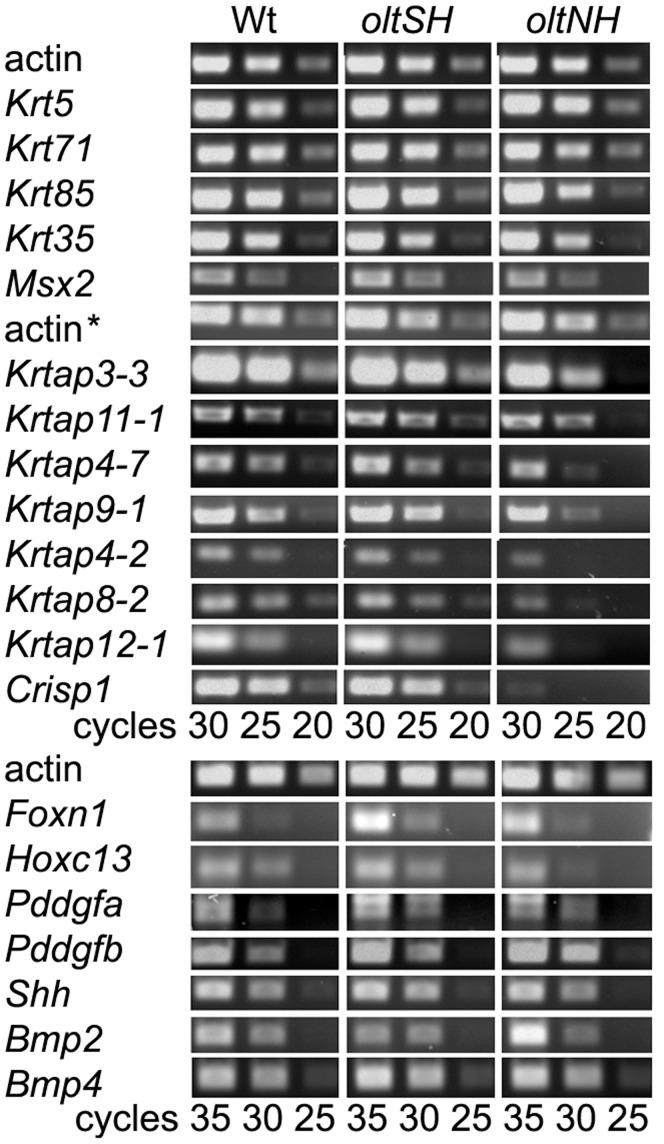
Expression of genes relevant to the developing hair follicle. Total RNA or mRNA was prepared from dorsal skin biopsies of postnatal day 8 of wild-type (*Del(9)olt1Pas* heterozygous and *Plcd3^mNab^* heterozygous), *oltSH* and *oltNH* mice and gene-specific fragments were amplified by PCR from 1 µg of cDNA. The number of PCR cycles is given below the image. The primers are listed in Table S2. The first strand was synthesised from mRNA preparations for all RT-PCR experiments with *Krtap* genes and actin* in the Figure. The limitation of PCR cycles gives a semi-quantitative estimate that *Foxn1*, *Msx2*, *Hoxc13*, *Pdgfa*, *Pdgfb*, *Shh*, *Bmp2* and *Bmp4*, as well as the hair keratins Krt35 and Krt86, and the IRS keratin (*Krt71*) are expressed at comparable levels in wild-type, *oltSH* and *oltNH* mutants on postnatal day 8, when the hair shaft in the specimens is being formed (see Figure 4C and 4I). However, *Crisp1* and *Krtap12-1*, and possibly also *Krtap4-2* and *Krtap8-2* are expressed at lower levels in *oltNH* mice compared to wild-type and *oltSH* mutants.

**Figure 11:**
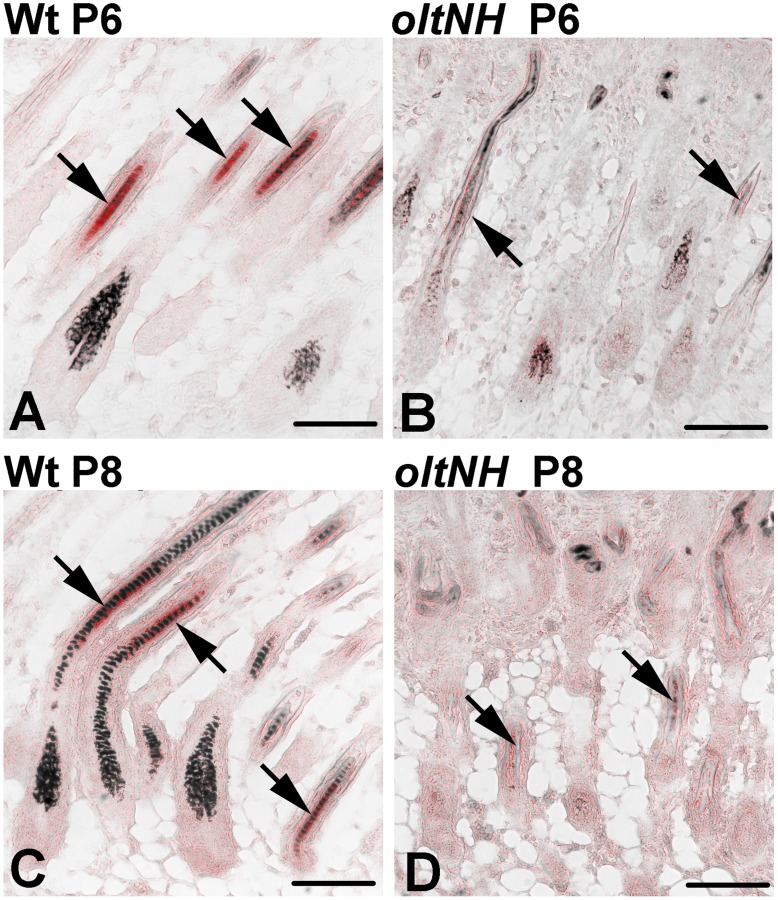
*Crisp1* expression in wild-type and *oltNH* hair follicles. In situ hybridisation with a gene-specific probe for *Crisp1* on skin sections of wild-type (A and C) and *oltNH* (B and D) mice on postnatal days 6 (A and B) and 8 (C and D). The DIG-labelled probe was visualised using alkaline phosphatase-conjugated anti DIG antibody. Images were taken in brightfield mode with a Nuance VX camera and further processed by spectral analysis using the accompanying software. The specific in situ signal spectrum was pseudo-coloured in red and the eumelanin spectral signal in black. Three biopsies from different animals were used for each time point investigated. Bar  = 100 µm. *Crisp1* expression is detected in the medulla of wild-type hair follicles (arrows in A and C, red signal), but not in comparable sections of *oltNH* mice (arrows in B and D).

To elucidate which cellular mechanisms might underlie the histological characteristics associated with the *oltSH* and *oltNH* mutations, we also investigated the expression of some genes involved in the growth and differentiation of the hair follicle as well as other genes encoding structural proteins of the outer and inner root sheath, and the hair shaft (Table S2). Semi-quantitative RT-PCR analysis on postnatal day 8 revealed no striking differences between wild-type, *oltSH* and *oltNH* dorsal skin hair follicles with respect to the expression of genes encoding several structural proteins like epidermal and outer root sheath keratin *Krt5*, hair shaft keratins *Krt85* and *Krt35*, IRS keratin *Krt71* and keratin associated proteins (*Krtap11-1*, *Krtap3-3*, *Krtap4-7, Krtap9-1*) [59]–[63]. Three important transcription factors involved in the transcription of hair keratin and keratin associated protein encoding genes in mice and humans [51], [64]–[74], *Foxn1, Msx2* and *Hoxc13*, showed unaltered expression between wild-type and *oltSH* and *oltNH* mutants. The expression levels of genes encoding for secreted signalling proteins *Pdgfa*, *Pdgfb*, *Shh*, *Bmp2* and *Bmp4* were also unchanged (Figure 10) [75]–[81]. However, *Krtap12-1* (in the hair cuticle) and *Crisp1* (in the hair medulla) [82] were clearly expressed at lower levels (Figure 10). In situ hybridisations using a gene-specific probe revealed that *Crisp1* is expressed in the medulla of the hair shaft in wild-type, but not *oltNH* mice on postnatal days 6 and 8 (Figure 11) confirming our RT-PCR data.

## Discussion

In this report, we demonstrate that mice lacking expression of both functional *Plcd1* due to a genomic deletion in the *Del(9)olt1Pas* mutant and functional *Plcd3* due to a genomic insertion of an IAP show total alopecia, weight loss and die during the first two months of life. While *Del(9)olt1Pas* homozygous mutant mice show a mild predominantly ventral alopecia due to the deletion of the *Plcd1* gene [47], homozygotes of the *Plcd3^mNab^* mutant allele have no obvious phenotype, which has also been described for mice in which *Plcd3* was inactivated by homologous recombination [38]. As experimental inactivation of both genes by homologous recombination causes early prenatal death with defects in the placenta [38], the novel *Plcd3^mNab^* allele described in this report behaves like a hypomorphic mutation of the *Plcd3* gene allowing for a limited period of postnatal life. While in *oltNH* mutants the bulk of *Plcd3* transcripts are shorter and immunblots predominantly show a truncated PLCD3 protein in *oltNH* mice, apparently normal transcripts of *Plcd3* can also be amplified by RT-PCR using primers in exon 1 and exon 5 (not shown). Therefore, we cannot exclude that trace amounts of normal transcript, too little to be detected in Northern blots, are expressed from the mutant *Plcd3^mNab^* allele, just sufficient to overcome the placental defects of the double knockout mice. Still, the *oltNH* mutant offers the unique opportunity to study postnatal functions of *Plcd1* and *Plcd3*.

Many spontaneous mouse mutations are the result of insertions of retroviral elements, recently reviewed in [48], [49], [83]. Subtypes of IAPs are particularly active in the C3H/HeJ strain of mice [84], which is related to the genomic background of the *Del(9)olt1Pas* mutant mice, in which the *Plcd3^m1Nab^* mutation occurred. Similar IAP insertions with 98% sequence homology to the *Plcd3^m1Nab^* IAP have recently been described in various mutant mice [57]. Due to the promoter and enhancer elements in the viral LTRs, such insertions into the genome can either increase or decrease the expression of adjacent host genes, or enhance the expression from endogenous promoters, as well as cause aberrant splicing [48], [49]. As in the case of the *Plcd3^m1Nab^* mutation, the IAP is integrated in reverse orientation with respect to the transcription of the *Plcd3* gene, it is most likely that it causes its effects by enhancing transcription from cryptic promoters. The *Plcd3* transcript variant ENSMUST00000128650 starting in exon 5 would be predicted to have a molecular weight well within the range of the *oltNH* mutant PLCD3 protein detected in our immunoblots. This truncated PLCD3 protein would have no PH domain, which is important for the targeting of the enzyme to the substrate in the plasma membrane [23], [25].

Mutant mice homozygous for the *Del(9)olt1Pas* mutation [47], *oltSH* and *oltNH* mutants (this report) exhibit a similar histological aspect of fragile hair shafts, but differ with respect to the distribution of this defect over the body surface. Similar fragility of hair shafts has been found in mice with altered expression of *Foxn1* and *Hoxc13*, which are directly involved in the expression of hair keratins and keratin associated proteins [60], [61], [64]–[73], [85], [86]. The co-expression of *Foxn1* and *Plcd1* in the pre-cortical zone of the hair bulb and the down-regulation of *Plcd1* in nude mice suggested that *Plcd1* has some function downstream of *Foxn1* [46], [68], [73]. We found that the expression domain of *Plcd3* encompasses the entire hair bulb during anagen including and exceeding the domain of *Plcd1*. It is therefore possible that *Plcd3* can compensate for some aspect of *Plcd1* function. Since we demonstrated that on postnatal day 8 the medulla-specific, *Hoxc13*-regulated gene *Crisp1* is down-regulated in *oltNH* mice, but not in *oltSH* mice, some hair shaft-specific genes may depend strictly on the expression of *Plcd3.* This may imply that in the absence of normal *Plcd1* expression, *Plcd3* could possibly play a role in the *Hoxc13*– *Foxq1* axis of gene regulation in the hair medulla [82], [86].

It has previously been suggested that the alopecia of mice with functional inactivation of *Plcd1* develops in the context of an inflammatory response [44]. We also observed neutrophilic granulocytes in *oltNH* skin, but also in *oltSH* mutants, in which at least some hair follicles were apparently in anagen. As the infiltrates in day 12 *oltSH* skin were associated with hair follicles in premature catagen, the influence of the inflammatory response is possibly very locally elicited and operative, but may contribute to the sustenance of the abnormal hair follicle regression in the mutant.

Recently, *Plcd1* was shown to exert direct effects on adipocytes. Knockdown of *Plcd1* in an adipocyte cell line interfered with lipid accumulation during differentiation, which was also observed in primary cells obtained from *Plcd1* defective mice [87]. This serves to explain the reduced body mass in mice lacking *Plcd1* expression. Further studies will reveal whether *Plcd3* is also involved in adipocyte differentiation and function in order to explain, why *oltNH* mice show an even more dramatic reduction in body mass than mice lacking functional *Plcd1* alone. Inflammatory infiltrates in *oltNH* mice were usually found in the subcutaneous adipose tissue near the dermis, where adipocyte precursors are located. Most recently, the stimulatory activity of BMP (bone morphogenetic protein) and PDGFA (platelet-derived growth factor A) secreted by subcutaneous fat cells and their precursors, respectively, has been highlighted with respect to their stimulating activity for hair follicle stem cells and the sustenance of anagen [88]. The inflammatory response in the subcutaneous adipose layer of *oltNH* mice could possibly negatively interfere with the stimulatory activity of this tissue, which could contribute to the curtailment of the growth phase in the mutant hair follicle.

Phospholipase C delta isozymes are associated with signal transduction processes involved in cell cycle regulation and cell proliferation [2], [7], [30], [31], [35], [89]–[92], and are altered in various tumours and tumour cell lines [93]–[97]. PLCD1 protein can also translocate to the nucleus and exert direct effects in the nuclear compartment [6], [7], [33], [34], [98]. We found that growth in the hair follicle bulb is uniformly short-phased in the *oltNH* mutant after birth and ceases on postnatal day 12, while anagen was less shortened in the lower ventral body region of *Plcd1* defective *Del(9)olt1Pas* mutants, where the alopecia of this mutant is most pronounced [47]. Since *Plcd3* is expressed in the hair bulb during this critical period, *Plcd1* and *Plcd3* may possibly play additive roles in the sustained growth during hair follicles morphogenesis after birth.

## Materials and Methods

### Ethics Statement

The animals were sacrificed according to §4.3 of the German law for the protection of animals (Tierschutzgesetz) (file number 50.203.2-BN 6/02). The mice were killed by cervical dislocation avoiding unnecessary pain.

### Mice

We have previously described the origin of *Del(9)olt1Pas* mutant mice (synonym *Del(9Ctdspl-Slc22a14)1Pas,* formerly called oligotriche, *olt*) [47]. The *oltSH* and *oltNH* mice were in a mixed C3He/Orl, C3H/HeJ and C3Heb/FeJ background, which we collectively refer to as C3H in this report. The animals were kept in a 12 hour light/dark cycle with food and water ad lib.

### Histology, Immunohistochemistry and In Situ Hybridisation

Skin biopsies were taken from the dorsal mid-thoracic region of mice after killing them by cervical dislocation. The skin biopsies were fixed for 4 hours in Bouin’s solution, dehydrated in increasing alcohol concentrations and embedded in paraffin as described [47]. Histological staining with haematoxylin/eosin and immunohistochemistry with mouse monoclonal antibodies against PCNA (proliferating cell nuclear antigen) (Bio-Genex, MU252-KL) diluted 1∶200 and Cy3-conjugated goat anti mouse IgG F(ab)2 (Dianova 115-165-672) diluted 1∶800 were performed as described [47].

The TUNEL assay was performed on paraffin sections using the Apoptag Red In Situ Apoptosis Detection Kit (Millipore Chemicon, S7165) according to the manufacturer’s recommendations. Nuclei were stained with DAPI.

Tissue sections of three different biopsies of dorsal skin of wild-type and *oltNH* mice were analysed histomorphometrically with respect to the length of the hair follicle (from the distal end to the infundibulum) and the width of the hair bulb at its widest diameter. 50 measurements of both parameters were taken from each biopsy using the ImageJ software and statistically analysed with the paired t test using PrismGraphPad 4 software.

### PCR and RT-PCR

PCR experiments were performed in a TProfessional Basic Thermocycler (Biometra). Usually 35 cycles were run with the optimal annealing temperature chosen according to the recommendation of the supplier of Phusion high fidelity DNA polymerase (Thermo Scentific, Finnzymes, Finland). All primers (Tables S1 and S2) were purchased from Eurofins MWG BIOTECH AG (Ebersberg, Germany). PCR products were sequenced by Eurofins MWG BIOTECH AG, Ebersberg, Germany.

For RT-PCR experiments, the cDNAs were synthesised from total RNA (RNeasy Midi Kit, QIAGEN, Hilden, Germany) or mRNA (Oligotex mRNA mini kit, QIAGEN) from skin biopsies using PowerScript™ reverse transcriptase from CLONTECH (Palo Alto, CA) with an Oligo (dT)15 primer. PCR was performed on templates of cDNA or genomic DNA prepared from tail biopsies (DNeasy Mini Kit, QIAGEN, Hilden, Germany) using Phusion high fidelity DNA polymerase (Finnzymes, Finland) according to the manufacturer’s recommendations.

The cDNA preparations synthesised from mRNA were used to amplify expressed sequences of genes that have only one exon, e.g. some *Krtap* genes. Gene-specific fragments were amplified from 1 µg of cDNA with the optimal annealing temperature chosen according to the recommendation of the supplier of Phusion high fidelity DNA polymerase (Thermo Scientific, Finnzymes, Finland). The number of PCR cycles run was usually 35, or is otherwise given in the Figure legend.

### RNA Blot Hybridisation

Gel electrophoresis of RNA, blotting and detection of DIG labelled probe was performed as described previously [47].

To synthesize DIG labelled cRNA probes, RT-PCR products were re-amplified using a modified antisense primer, to which the sequence of the T7 RNA polymerase binding site (GGATCCTAATACGACTCAC) had been added at its 5′ end. Antisense cRNA probes were then derived from these PCR products by *in vitro* transcription in the presence of DIG-labelled dUTPs with T7 RNA polymerase from Roche (Mannheim, Germany) [47].

The cRNA probe for Northern blot hybridisations to *Plcd3* mRNA was generated from skin cDNA using primers 511 (exon 11) und 512 (3′UTR) (Table S1). DIG labelled probes for RNA blot hybridisations were synthesised by PCR using the PCR DIG probe synthesis kit (Roche). The cRNA probe for *Plcd1* has been described [47].

### In Situ Hybridisation

The 350 b *Plcd3-*specific cRNA probe was generated as described above from the RT-PCR product synthesised with forward primer 527 (exon 3) and reverse primer 1352 (exon 5) (Table S1) and the 400 bp Crisp1 probe was generated in the same fashion using forward primer FR634 and reverse primer FR635 (Table S2). In situ hybridisations were performed as described [47].

Because the histochemical stain for alkaline phosphatase activity in Bouin-fixed tissue sections gives a rather brownish colour and is difficult to distinguish from pigmented areas, skin biopsies were taken from C57BL/6J mice that have mostly black eumelanin and the sections were photographed and analysed using a NuanceVX multispectral camera (obtained from INTAS, Goettingen, Germany) with the manufacturer’s software. Areas displaying spectral characteristics of eumelanin were pseudo-coloured in black, those displaying spectral characteristics of the specific in situ signal in red.

### DNA Blot Hybridisation

Genomic DNA was digested with BamHI (Fermentas), the fragments separated by agarose gel electrophoresis and transferred to positively charged Nylon membrane (Roche) by vacuum blotting using a Model 785 Vacuum Blotter (BIORAD). Hybridisation was carried out in Dig Easy Hyb (Roche) using 100 ng/ml of DIG labelled probe. Washes and detection of bound DIG were carried out as described in the Roche DIG Application Manual for Filter Hybridisations (https://www.roche-applied-science.com/PROD_INF/MANUALS/DIG_MAN/dig_toc.htm).

DIG labelled probes for DNA blot hybridisations were synthesised by amplifying genomic fragments using the PCR DIG probe synthesis kit (Roche). For hybridisations to *Plcd3*-specific genomic fragments, we amplified a 900 bp fragment of intron 1, 800 bp upstream of exon 2, using primers 572 and 573 (Table S1) (marked as P in Figure 2A).

### Western Blot

Mouse monoclonal antibodies binding specifically to the catalytic domain of the PLCD3 protein (IF12–15) or the PH domain of PLCD3 (4H 5–9), respectively, were supplied by K. Fukami. The hybridoma supernatant was used undiluted. The secondary antibody, (peroxidase-conjugated goat anti mouse Ig, Dianova) was used at a dilution of 1∶30.000. Blotting and signal detection were performed as described [47].

## Supporting Information

Figure S1 **Origin of** ***oltNH*** **mice.** Pedigree showing the breeding scheme that led to the discovery of *oltNH* mice. White symbols represent wild-type mice and blue symbols mice with the phenotype of *Del(9)olt1Pas* homozygotes. Red symbols represent mice of the *oltSH* phenotype and yellow symbols are mice of the *oltNH* phenotype. The symbols merely show the presence of such phenotype in a litter, but do not represent the relative proportion of phenotypes in each litter. The numbers designate the crosses referred to in the following text. All mice in the F_1_ generation of a *Del(9)olt1Pas* female mated with a wild-type C3HeB/FeJ male (cross 1) were phenotypically normal. In the F_2_ generation of cross 2, (56 mice in 7 litters), we found 10 phenotypically *Del(9)olt1Pas* homozygotes and one female that showed a greater extent of alopecia, which we termed *oltSH* (for sparse hair). This female was backcrossed with her father (cross 3) resulting in one litter of six, in which 2 of 6 mice showed the *oltSH* phenotype. When one of these *oltSH* females was crossed with a known *Del(9)olt1Pas* heterozygous male (cross 4), the phenotypically altered offspring showed in equal parts the *oltSH* and the *Del(9)olt1Pas* homozygous phenotype, suggesting that one dose of a novel mutation could exacerbate the alopecia in *Del(9)olt1Pas* homozygous mice. When the *oltSH* founder female of the F_2_ generation was crossed with one of the phenotypically normal sons in cross 5, we found altogether 52 mice with different phenotypic alterations in the offspring (n  = 121 in 21 litters): the *Del(9)olt1Pas* mutant phenotype, the *oltSH* phenotype, but also 10 mice that did not developed any visible pelage and only a few short vibrissae, which we termed *oltNH*. Further brother-sister matings of this offspring (cross 6) again produced all three mutant phenotypes.
(TIF)

Table S1 **List of** ***Plcd3-*** **specific primers.** The oligonucleotide sequences are given in 5′ to 3′ orientation; genome coordinates are given according to NCBI37 mm9 July 2007; “*Plcd3* genomic” indicates the location of the oligonucleotide sequence in the *Plcd3* locus.
(DOCX)

Table S2 **List of primers used in** **Figure 10** **.** The oligonucleotide sequences are given in 5′ to 3′ orientation. (F, forward, R, reverse)
(DOC)
