# Supplementary figures and images for: Alopecia in a Viable Phospholipase C Delta 1 and Phospholipase C Delta 3 Double Mutant
Source: PLoS One. 2012 Jun 19;7(6):e39203. doi: 10.1371/journal.pone.0039203 (PMC3378570; doi:10.1371/journal.pone.0039203)

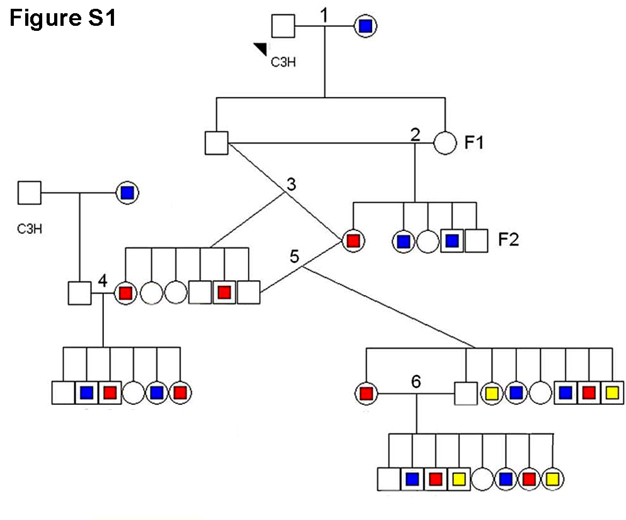

Supplement: Figure S1 — Origin of oltNH mice. Pedigree showing the breeding scheme that led to the discovery of oltNH mice. White symbols represent wild-type mice and blue symbols mice with the phenotype of Del(9)olt1Pas homozygotes. Red symbols represent mice of the oltSH phenotype and yellow symbols are mice of the oltNH phenotype. The symbols merely show the presence of such phenotype in a litter, but do not represent the relative proportion of phenotypes in each litter. The numbers designate the crosses referred to in the following text. All mice in the F1 generation of a Del(9)olt1Pas female mated with a wild-type C3HeB/FeJ male (cross 1) were phenotypically normal. In the F2 generation of cross 2, (56 mice in 7 litters), we found 10 phenotypically Del(9)olt1Pas homozygotes and one female that showed a greater extent of alopecia, which we termed oltSH (for sparse hair). This female was backcrossed with her father (cross 3) resulting in one litter of six, in which 2 of 6 mice showed the oltSH phenotype. When one of these oltSH females was crossed with a known Del(9)olt1Pas heterozygous male (cross 4), the phenotypically altered offspring showed in equal parts the oltSH and the Del(9)olt1Pas homozygous phenotype, suggesting that one dose of a novel mutation could exacerbate the alopecia in Del(9)olt1Pas homozygous mice. When the oltSH founder female of the F2 generation was crossed with one of the phenotypically normal sons in cross 5, we found altogether 52 mice with different phenotypic alterations in the offspring (n = 121 in 21 litters): the Del(9)olt1Pas mutant phenotype, the oltSH phenotype, but also 10 mice that did not developed any visible pelage and only a few short vibrissae, which we termed oltNH. Further brother-sister matings of this offspring (cross 6) again produced all three mutant phenotypes. (TIF) [file pone.0039203.s001.tif]
