# Supplementary material for: Alopecia in a Viable Phospholipase C Delta 1 and Phospholipase C Delta 3 Double Mutant
Source: PLoS One. 2012 Jun 19;7(6):e39203. doi: 10.1371/journal.pone.0039203 (PMC3378570; doi:10.1371/journal.pone.0039203)
Supplement: Table S1 — List of Plcd3- specific primers. The oligonucleotide sequences are given in 5′ to 3′ orientation; genome coordinates are given according to NCBI37 mm9 July 2007; “Plcd3 genomic” indicates the location of the oligonucleotide sequence in the Plcd3 locus. (DOCX) [file pone.0039203.s002.docx]

List of *Plcd3-*specific primers used.

| Name | Sequence | Genome | *Plcd3* genomic |
| --- | --- | --- | --- |
| 507 | GGTTCTCACGCACAGATCC | 102962872 - 102962890 | exon 1 |
| 1046 | CTCGCGCAGCTTCTAAGCAC | 102941822 - 102941841 | exon 2 |
| 900 | TGATGACCCTCCCGGACAGC | 102941690 - 102941709. | exon 3 |
| 1049 | GCCAGGCAGGATATTGGTC | 102941486 -102941505 | exon3 |
| 527 | TCTTCGTGCAGCACATCG | 102941710 - 102941729 | exon3 |
| 1352 | ACGCTCGTTGTTGGAATGGT | 102939248 - 102939267 | exon 5 |
| 511 | GCAACTGACCCGAGTGTACC | 102935111 - 102935130 | exon 11 |
| 512 | CTGAAAGGAGGCAGTGAAGG | 102931803 - 102931822 | 3’ UTR |
| 976 | GTTGGTGGCCAATTCTGAGC | 102942551- 102942570. | intron 1 |
| 962 | CATGCGCCACCACTGCAACC | 102943578 - 102943598 | intron 1 |
| 953 | CCCTGGGCTTGGCATCAGAGG | 102955953 - 102955973. | intron 1 |
| 572 | CCTGTAATGCCAGCACACC | 102943706 - 102943725. | intron 1 |
| 573 | TCCAGGGTCCGTATTTCTCC | 102942795 - 102942814 | intron 1 |
| 1400 | GTTCAAGATCAGACTTACCTCGTTCC |  | IAP 5’ LTR. |

The oligonucleotide sequences are given in 5’ to 3’ orientation; genome coordinates are given according to NCBI37 mm9 July 2007; “*Plcd3* genomic” indicates the location of the oligonucleotide sequence in the *Plcd3* locus.
