# Supplementary material for: Alopecia in a Viable Phospholipase C Delta 1 and Phospholipase C Delta 3 Double Mutant
Source: PLoS One. 2012 Jun 19;7(6):e39203. doi: 10.1371/journal.pone.0039203 (PMC3378570; doi:10.1371/journal.pone.0039203)
Supplement: Table S2 — List of primers used in Figure 10 . The oligonucleotide sequences are given in 5′ to 3′ orientation. (F, forward, R, reverse) (DOC) [file pone.0039203.s003.doc]

List of primers used in Figure 10

| Primer |  | Sequence | Gene and primer start in the cDNA (bp) |
| --- | --- | --- | --- |
| FR1021 | F | CTTTGCAGCTCCTTCGTTGC | *Actb*  ENSMUSG00000029580 bp 35 |
| FR1022 | R | GGTTGGCCTTAGGGTTCAGG | *Actb* ENSMUSG00000029580 bp 426 |
| FR1334 | F | ACGCCATTGCTGAAGCTGAG | *Krt5* ENSMUST00000023709 bp 1317 |
| FR1335 | R | CTTAGCCCGCTACCCAAACC | *Krt5*  ENSMUST00000023709 bp 1675 |
| FR443 | F | TCCAAGTGTTCTGAGCCTGGGTAGC | *Krt71* ENSMUST00000023710 bp 1784 |
| FR446 | R | AAAGAATACAAGAGAAGCCAAGG | *Krt71* ENSMUST00000023710 bp 2122 |
| FR1332 | F | CCGAGATCGAGAATGCCAAG | *Krt85* GI:31980831 bp 1148 |
| FR1333 | R | TGGCCCTGAACTCTTTCCTC | *Krt85* GI:31980831 bp 1703 |
| FR1330 | F | GGAGAAGGCATCCTCACTGG | *Krt35* ENSMUST00000103127 bp 322 |
| FR1331 | R | GGAGCAGCATCCACCTCAAC | *Krt35* ENSMUST00000103127 bp 793 |
| FR2395 | F | GGCGGTGACTTGTTTTCGTC | *Msx2* ENSMUST00000021922 bp 402 |
| FR2396 | R | CCTTAGCCCTTCGGTTCTGG | *Msx2* ENSMUST00000021922 bp 934 |
| FR1602 | F | CTTGCTTGGCACCAGTCTTC | *Krtap3-3* ENSMUST00000092700 bp 385 |
| FR1603 | R | AAACCCCAAACACCCAAAGA | *Krtap3-3* ENSMUST00000092700 bp 615 |
| FR038 | F | GCCATCATGTCTTTCAACTGC | *Krtap11-1*  ENSMUST00000171542 bp 69 |
| FR039 | R | GTTGCAATGCCAACTAATGC | *Krtap11-1* ENSMUST00000171542 bp 990 |
| FR1587 | F | TGAGGAGGGCTGTAGCCAAG | *Krtap4-7* ENSMUST00000055121 bp 106 |
| FR1588 | R | GAAGCTGGATCAAGGCATGG | *Krtap4-7* ENSMUST00000055121 bp 566 |
| FR028 | F | TGAACACAACTCCTCCCTCC | *Krtap9-1* ENSMUST00000093936 bp 39 |
| FR1344 | R | AGCCACAGGGCTCACAAC | *Krtap9-1* ENSMUST00000093936 bp 590 |
| FR660 | F | TGTTTGCAATTGTTCCGTGT | *Krtap4-2* ENSMUST00000058987 bp 695 |
| FR661 | R | TGTCATGGGGATTAAAAATGC | *Krtap4-2* ENSMUST00000058987 bp853 |
| FR1605 | F | ACGGCAGCTACTACGGAGGT | *Krtap8-2* ENSMUST00000053460 bp 58 |
| FR1606 | R | GAATCGGGAGAATCCATATCC | *Krtap8-2* ENSMUST00000053460 bp 191 |
| FR658 | F | CAGATGGGACACACTTGCTG | *Krtap12-1* ENSMUST00000092370 bp 477 |
| FR659 | R | AGCCAGGACATCCATGAGAG | *Krtap12-1* ENSMUST00000092370 bp 595 |
| FR633 | F | CCAGATGGAGAAACCATTCG | *Crisp1* ENSMUST00000026498 bp 390 |
| FR634 | R | CTTGCATCATGGTCTTCTGC | *Crisp1* ENSMUST00000026498 bp 749 |
| FR2401 | F | GCCCTCAATCCTTCCAAAAT | *Foxn1* ENSMUST00000108294 bp 1151 |
| FR458 | R | AGGTCAGTCCCAAGGTCTCC | *Foxn1* ENSMUST00000108294 bp 1660 |
| FR2398 | F | GGTGACGACCTGTCCTCCAG | *Hoxc13* ENSMUST00000001700 bp582 |
| FR2399 | R | TTCGGGTGGATTCCGTTATG | *Hoxc13* ENSMUST00000001700 bp1138 |
| FR2368 | F | AGACTCCGTAGGGGCTGAGG | *Pdgfa* ENSMUST00000046901 bp 351 |
| FR2369 | R | GTCTCCTCCTCCCGATGGTC | *Pdgfa* ENSMUST00000046901 bp 741 |
| FR2370 | F | TCTGCTGCTACCTGCGTCTG | *Pdgfb*  ENSMUST00000000500 bp 861 |
| FR2371 | R | AGATGGGCTTCTTTCGCACA | *Pdgfb* ENSMUST00000000500 bp 1284 |
| FR2403 | F | GCTGCTGGCCAGATGTTTTC | *Shh* ENSMUST00000002708 bp 366 |
| FR2404 | R | CGTGGTGATGTCCACTGCTC | *Shh* ENSMUST00000002708 bp 772 |
| FR2406 | F | AGGCTGAGGGATGCAGAGTG | *Bmp2* ENSMUST00000028836 bp 632 |
| FR2407 | R | CCTCCTTCTCCGGGTGTTCT | *Bmp2* ENSMUST00000028836 bp 1044 |
| FR2409 | F | AACCGAATGCTGATGGTCGT | *Bmp4* ENSMUST00000074077 bp 530 |
| FR2410 | R | CTCTGGGATGCTGCTGAGGT | *Bmp4* ENSMUST00000074077 bp 929 |

The oligonucleotide sequences are given in 5’ to 3’ orientation. (F, forward, R, reverse)
